# Supplementary material for: Wing mechanics and acoustic communication of a new genus of sylvan katydid (Orthoptera: Tettigoniidae: Pseudophyllinae) from the Central Cordillera cloud forest of Colombia
Source: PeerJ. 2024 Jun 28;12:e17501. doi: 10.7717/peerj.17501 (PMC11216201; doi:10.7717/peerj.17501)
Supplement: Supplemental Information 1 [file peerj-12-17501-s001.pdf]

Since our initial submission we have made the decision to invite a new author onto our paper, Dr Emine Celiker. Dr Celiker was not involved in the initial submission as we had no need for a numerical acoustic analysis. However, the decision was made to invite her following a comment made by the third reviewer who questioned the reliability of using ABS-like resin and the compliance of the tympanal membranes in our 3D printed models. Dr Celiker is an expert mathematician in numerical modelling of animal ears who developed a method to handle the katydid 3D ear geometries for numerical acoustic analysis, which allowed us to provide an accurate response on the reliability of our 3D models. This is not our field of expertise and is a huge contribution to the paper.

Emine started by creating two numerical models: The first one incorporated realistic material properties of *S. sodalis* pinnae and tympana, and the second simulation incorporated the material properties of grey ABS-like photopolymer resin for the whole cavity geometry, as used during 3D printing. She wrote the methods for this process which can be found at lines 251-278. Thanks to Emine, we show that there is very little difference in resonant frequency and acoustic gain between the two models, thus highlighting the reliability and accuracy of our 3D printed models. She went on to describe our findings in the results section (lines 654-664) and the discussion (lines 720-730). In addition to this she created the figures 17B; 17C; 17D.

Without Dr Celiker we would have been unable to provide such a detailed response to the reviewers query. All previous authors have agreed to her involvement and her additions to the manuscript have been made clear in the resubmission.
